# Lymph Node-Targeting Nanovaccine through Antigen-CpG Self-Assembly Potentiates Cytotoxic T Cell Activation

**DOI:** 10.1155/2018/3714960

**Published:** 2018-06-19

**Authors:** Xiaobo Xi, Lijun Zhang, Guihong Lu, Xiaoyong Gao, Wei Wei, Guanghui Ma

**Affiliations:** ^1^State Key Laboratory of Biochemical Engineering, Institute of Process Engineering, Chinese Academy of Sciences, Beijing 100190, China; ^2^University of Chinese Academy of Sciences, Beijing 100049, China; ^3^School of Life Science, Beijing Institute of Technology, Beijing 100081, China; ^4^Jiangsu National Synergetic Innovation Center for Advanced Materials, Nanjing 211816, China

## Abstract

Therapeutic vaccines that arouse the cytotoxic T cell immune response to reject infected cells have been investigated extensively for treating disease. Due to the large amounts of resident antigen-presenting cells (APCs) and T cells in lymph nodes, great efforts have been made to explore the strategy of targeting lymph nodes directly with nanovaccines to activate T cells. However, these nanovaccines still have several problems, such as a low loading efficiency and compromised activity of antigens and adjuvants derived from traditional complicated preparation. There are also safety concerns about materials synthesized without FDA approval. Herein, we construct an assembled nanoparticle composed of an antigen (ovalbumin, OVA) and adjuvant (CpG) to ensure its safety and high loading efficiency. The activity of both components was well preserved due to the mild self-assembly process. The small size, narrow distribution, negative charge, and good stability of the nanoparticle endow these nanovaccines with superior capacity for lymph node targeting. Correspondingly, the accumulation at lymph nodes can be improved by 10-fold. Subsequently, due to the sufficient APC internalization and maturation in lymph nodes, ~60% of T cells are stimulated to proliferate and over 70% of target cells are specifically killed. Based on the effective and quick cellular immune response, the assembled nanoparticles exhibit great potential as therapeutic vaccines.

## 1. Introduction

Traditional prophylactic vaccines, which act via humoral immunity, fail to combat infected or neoplastic cells [1, 2]. Much effort has been devoted to develop cellular immunity-mediated therapeutic vaccines [3]. During the cellular immune response, cytotoxic T cells play a central role in eliminating target cells [4, 5]. To activate cytotoxic T cells, antigens should be captured and presented via the major histocompatibility complex- (MHC-) I [6]. However, antigen alone with rapid clearance shows little effect on the cellular response [7]. Therefore, strategies for efficient antigen internalization and subsequent MHC-I presentation are urgently needed for therapeutic vaccines.

Inspired by the aluminum adjuvant widely used in prophylactic vaccines, researchers have developed various systems to serve as the antigen depot at the vaccination site [8, 9]. Along with the sustained release of antigen within several days in the retention systems, a flow of antigen-presenting cells (APCs) can be recruited for uptake and maturation, and these then home to lymph nodes to activate T cells. During this process, many factors are responsible for the generated immune responses, such as number and type of recruited APCs, uptake amount of antigen, and APC maturation and subsequent trafficking to lymph nodes [10–12]. As a large number of APCs and T cells reside in lymph nodes, directly delivering antigen into lymph nodes is being considered as an alternative and even preferred strategy [13, 14]. Correspondingly, many nanodelivery systems are being developed, including polymer and inorganic nanoparticles, liposomes, dendrimers, and micelles [15, 16]. In addition to antigen, adjuvants such as CpG oligodeoxynucleotides (CpG ODN) and flagellin can be codelivered to the lymph nodes, which will significantly improve the MHC-I presentation for cytotoxic T cell activation [13, 17]. Although promising, these nanovaccines still have several problems. In most cases, antigen and adjuvant are loaded via encapsulation or conjugation [18, 19]. The harsh process that this involves, such as homogenization and use of an organic solvent, can compromise their activity and loading efficiency [20, 21]. Moreover, most synthesized materials utilized as the framework of nanovaccines have yet been approved by the United States Food and Drug Administration (FDA) [22]. Their biosafety remains a critical issue to resolve before their clinical use. Therefore, developing a facile approach for constructing safe and high-performance nanovaccines with satisfactory payloads is still necessary and challenging.

**Figure 1:**
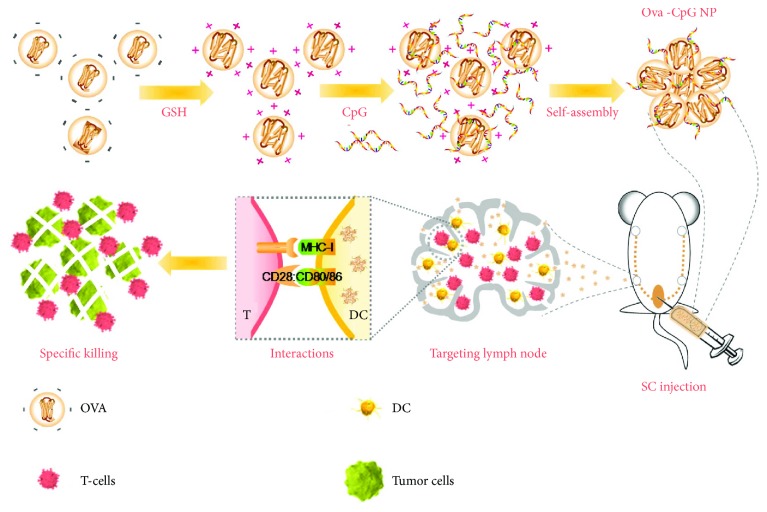
A schematic diagram of OVA-CpG NP fabrication and vaccine immunization strategy. First, GSH was added to change the charge of OVA to positive. Then, an electrostatic interaction could be triggered with the further addition of negatively charged CpG, and the OVA-CpG NPs could self-assemble. After subcutaneous (sc) injection, OVA-CpG NPs drained to lymph nodes and stimulated the maturation of DCs. Then, an effective T cell immune response was aroused to kill tumor cells.

Keeping this in mind, we developed lymph node-targeting nanovaccines through antigen-CpG self-assembly for cytotoxic T cell activation (Figure 1). In a typical preparation, GSH was first utilized to break up intramolecular disulfide bonds of antigen (ovalbumin (OVA)) and reverse the molecular charge to positive. With further addition of CpG adjuvant, the electrostatic interaction triggered the self-assembly process for the formation of OVA-CpG NP. After immunization, these nanovaccines quickly and efficiently drained to lymph nodes for APC internalization and maturation. As a result, proliferation of cytotoxic T cells and their specific lysis of target cells were significantly enhanced, demonstrating the potential use of this technology for therapeutic vaccinations.

## 2. Materials and Methods

### 2.1. Materials and Reagents

Model antigen OVA was purchased from Sigma-Aldrich (St. Louis, MO, USA), CpG 5′-TCCATGACGTTCCTGACGTT-3′). FAM-labeled CpG and control ODN (5′-TCCATGAGCTTCCTGAGCTT-3′) were obtained from Sangon (Shanghai, China). Carboxyfluorescein diacetate succinimidyl ester (CFSE) dye and the Micro BCA protein assay kit (BCA) were purchased from Thermo Fisher Scientific (Waltham, MA, USA). Fluorochrome-conjugated anti-mouse antibodies were obtained from eBioscience (San Diego, CA, USA) and BioLegend (San Diego, CA, USA). Recombinant mouse GM-CSF and IL-4 were obtained from PeproTech (Rocky Hill, NJ, USA). Cy5-SE was purchased from Fanbo Biochemicals Company (Beijing, China). GSH, ethanol, and NaCl were all of analytical grade. The medium for culturing dendritic cells, splenocyte cells, and tumor cells was RPMI 1640/DMEM (Gibco, Carlsbad, CA, USA) supplemented with 10% (*v*/*v*) fetal bovine serum (Gibco, Carlsbad, CA, USA), 100 U/mL penicillin (Invitrogen), and 100 *μ*g/mL streptomycin (Invitrogen).

### 2.2. Cell Lines and Animals

EL4 and E.G7-OVA (derivative of EL4) cells were provided by the State Key Laboratory of Biochemical Engineering (Beijing, China). OT-I mice used were provided by the State Key Laboratory of Biochemical Engineering (Beijing, China). Female C57BL/6 mice used in this study were purchased from Vital River Laboratories (Beijing, China). All animal experiments were performed in accordance with the Guide for the Care and Use of Laboratory Animals and were approved by the Experimental Animal Ethics Committee in Beijing.

### 2.3. Preparation and Characterization of Vaccine Formulations

OVA was dissolved in 1 mL 0.1 M NaCl solution with GSH (2 mM, pH = 9.6). The mixed solution was stirred to break intramolecular disulfide bonds. Then, CpG ODN was slowly added to the solution to precipitate the OVA-CpG nanoparticles (NPs). The suspension was kept under stirring for 30 min and ultracentrifuged to collect nanoparticles (200000*g*, 60 min, Figure S1). After that, the precipitate was washed three times with deionized water to remove GSH, and the concentration of OVA in the collected supernatant was measured by using a micro BCA protein assay kit. Finally, the nanoparticles were redissolved with distilled water and kept at 4°C, and the yield of nanoparticles was calculated using the equation below:

(1) $$\begin{array}{c} Yield\left(\%\right)=\left(1-\frac{mass\text{ of }OVA\text{ in }collected\;supernatant}{mass\text{ of }initial\;OVA}\right)\times 100\%. \end{array}$$

Control ODN was used to prepare OVA NPs. FAM-CpG was introduced to prepare OVA-FAM-CpG NPs, and the fluorescence intensity of free FAM-CpG in the supernatant was detected using a microplate reader (Infinite 2000, Tecan). Cy5-SE was added to react with the suspension of OVA and FAM-CpG to prepare Cy5-OVA-FAM-CpG NPs.

OVA-CpG NPs were observed by scanning electron microscopy (JEOL, Japan). Particle size and zeta potential in distilled water (pH ≈ 6.8) were measured by a Nano ZS Zetasizer in water (Malvern, UK). Cy5-OVA-FAM-OVA NPs were characterized by a CytoFLEX LX flow cytometer (Beckman Coulter, USA) and SP5 CLSM (Leica, Germany).

The far circular dichroism (CD) spectra (190–260 nm) of OVA and OVA in GSH solution were measured by a J-810 spectropolarimeter (JASCO, Japan) using a 1 mm path length cuvette at 25°C. The spectra of deionized water and 1 mM GSH solution were measured first as blanks and subtracted from the spectra of samples.

### 2.4. Antigen Uptake and Activation of Bone Marrow Dendritic Cells (BMDCs) In Vitro

BMDCs were derived using an established protocol. In brief, bone marrow cells were isolated from female C57BL/6 mice and cultured in RPMI 1640-based medium supplemented with GM-CSF and IL-4 for 6 days to harvest immature DCs. To evaluate antigen uptake, immature DCs were incubated with Cy5-OVA (10 *μ*g/mL), FAM-CpG (1 *μ*g/mL), Cy5-OVA NPS, or Cy5-OVA-FAM-CpG NPs for 12 h and collected. Cells were then washed clear, followed by staining with PE-CD11c antibody to identify DCs. The percentages of CD11c^+^OVA^+^, CD11c^+^CpG^+^, and CD11c^+^OVA^+^CpG^+^ cells were measured using a CytoFLEX LX flow cytometer (Beckman Coulter, USA) and analyzed with FlowJo software (version 7.6).

To evaluate the time-dependent uptake of OVA/CpG and OVA-CpG NPs in DCs, DCs cultured in a 24-well plate were incubated with Cy5-OVA/FAM-CpG and Cy5-OVA-FAM-CpG NPs for different time intervals until 24 h. In each group, 10 *μ*g soluble OVA or an equivalent dose of OVA NPs loaded with or without 1 *μ*g CpG was used. Then, cells were harvested and washed by PBS, and the uptake amount of Cy5-OVA and FAM-CpG was determined by FCM and analyzed using FlowJo software.

To evaluate the activation of DCs, BMDCs were challenged with PBS, OVA, OVA mixed with CpG (OVA/CpG), OVA NPs, OVA NPs mixed with CpG (OVA NPs/CpG), or OVA-CpG NPs for 24 h. In each group, 10 *μ*g soluble OVA or an equivalent dose of OVA NPs loaded with or without 1 *μ*g CpG was used. Antibodies against PE-CD11c, FITC-CD40, APC-Cy7-CD86, and eFluor 450-SIINFEKL (OVA-specific MHC-I peptide) were then added to stain the surface markers of DCs, and then the expression of the markers was measured by FCM and analyzed using FlowJo software.

### 2.5. Evaluation of Targeting Lymph Nodes

Female C57BL/6 mice aged 6–8 weeks were administered subcutaneously with Cy5-OVA or Cy5-OVA-CpG NPs. The clearance kinetics in situ and accumulation kinetics in lymph nodes of different vaccine formulations were monitored using in vivo imaging system FX Pro (Kodak) at different time points, and lymph nodes were extracted and scanned.

### 2.6. DC Uptake in Lymph Nodes

Female C57BL/6 mice aged 6–8 weeks were administered subcutaneously with PBS, Cy5-OVA, Cy5-OVA/FAM-CpG, Cy5-OVA NPs, or Cy5-OVA-FAM-CpG NPs. In each group, 20 *μ*g soluble OVA or an equivalent dose of OVA NPs loaded with or without 2 *μ*g CpG was used. The proximal lymph nodes at the injection sites were harvested at 8 h after immunization. The single-cell suspensions from lymph nodes were prepared by mechanical disruption and isolated from red cell using red blood cell lysis buffer. Cells were collected by centrifugation and stained with PE-CD11c for analyzing DCs. Then, the uptake amount of Cy5-OVA and FAM-CpG was determined by FCM and analyzed using FlowJo software.

### 2.7. Stimulation of CD8^+^ DCs In Vivo

To verify the activation ability of OVA-CpG NPs, mice were immunized with PBS, OVA, OVA-NP, OVA mixed with CpG (OVA/CpG), OVA NP mixed with CpG (OVA-NPs/CpG), or OVA-CpG NPs. In each group, 20 *μ*g soluble OVA or an equivalent dose of OVA NPs loaded with or without 2 *μ*g CpG was used. Draining lymph nodes were harvested after 24 h to prepare single-cell suspensions. Antibodies against PE-CD11c, APC-CD8, FITC-CD40, APC-Cy7-CD86, and eFluor 450-SIINFEKL (OVA-specific MHC-I peptide) were then added to stain the surface markers of DCs. The expression of related markers was measured by FCM and analyzed using FlowJo software.

### 2.8. T Cell Proliferation

To evaluate the proliferation of OVA-specific CD8 T cells in vivo, OT-I T cells were first separated and stained with CFSE (2 *μ*M). Then, 2 × 10^6^ CFSE-labeled OT-I T cells were administered intravenously before immunization. The mice were sacrificed at day 1 after immunization, and LN cells were harvested and stained with PE-CD3 and eFluor 450-CD8 antibodies. The proliferation of OVA-specific CD8^+^ T cells was assessed by FCM.

### 2.9. Cytotoxicity Activity of CTL

Spleens of immunized mice were extracted to evaluate the activity of antigen-specific CTL. The single-cell suspensions of splenocytes were stimulated with 5 *μ*g/mL SIINFEKL (OVA-specific MHC I) peptide for 3 days in RPMI 1640 medium supplemented with 20 U·mL^−1^ recombinant IL-2. Subsequently, the activated T cells were incubated with mitomycin-treated E.G7 cells or EL4 target cells. The CTL activity was evaluated at 5 : 1, 10 : 1, and 20 : 1 ratios of effector cells to target cells (E/T ratios) using a lactate dehydrogenase (LDH) cytotoxicity detection assay.

### 2.10. Statistical Analysis

All results are expressed as the mean ± sd. Unless otherwise noted, differences between two groups were evaluated using unpaired, two-tailed Student's *t*-test. Differences among more than two groups were evaluated by one-way ANOVA, with significance determined by Tukey-adjusted *t*-tests. Statistical significance was defined as ^∗^ *P* < 0.05 and ^∗∗^ *P* < 0.01.

## 3. Results and Discussion

### 3.1. Characterization of OVA-CpG NPs

**Figure 2:**
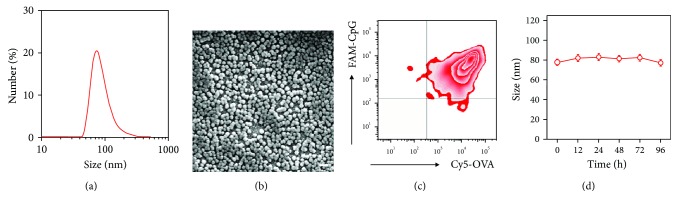
Characterization of OVA-CpG NPs. (a) Size distribution and SEM image (b, scale bar: 100 nm) of OVA-CpG NPs. (c) FCM image of OVA-CpG NPs (OVA was labeled with Cy5 and CpG was labeled with FAM). (d) Stability of OVA-CpG NPs in PBS. The bars represent sd (*n* = 3).

To enable the self-assembly, we first utilized GSH to break up intramolecular disulfide bonds of OVA [23]. During this mild process, the secondary conformation of OVA changed significantly (indicated by the circular dichroism of OVA in Figure S2). In this case, the OVA molecule charge reversed to positive, which might be attributed to the exposure of basic amino acids (Lys, Arg, and His). With further addition of negatively charged CpG adjuvant, the electrostatic interaction could then trigger the self-assembly process for the formation of OVA-CpG NPs. After optimization, the diameter of obtained OVA-CpG NPs was ~80 nm (Figure 2(a)) in the hydration microenvironment. During the dehydration process for SEM sample preparation, the NPs became shrunken. As a result, the particle size apparently decreased to ~50 nm (Figure 2(b)). Considering the size of individual OVA (~5 nm, Figure S3), we propose that each NP was composed of ~1000 OVA molecules. Meanwhile, the zeta potential of these NPs returned to negative (Figure S4), indicating a large amount of CpG had interacted with OVA (Figure S5). After calculation, the loading efficiencies of OVA and CpG were calculated up to 91.1% and 8.9%, respectively (Figure S6). These two dominant components were further verified by the colocalization of OVA and CpG as characterized by FCM and CLSM (Figure 2(c), Figure S7). Although such a structure was formed via electrostatic interaction, these NPs had little change on the particle size during storage in PBS (Figure 2(d)). The aforementioned features of OVA-CpG NPs together enabled them to serve as nanovaccines for cytotoxic T cell activation. Their small size, narrow distribution, and negative charge endowed them with the capacity for lymph node targeting, while the good stability ensured their integrity before the arrival at lymph nodes. The adequate payloads were also favored for the sufficient APC internalization and subsequent MHC-I presentation.

### 3.2. Enhanced APC Uptake and Maturation In Vitro

**Figure 3:**
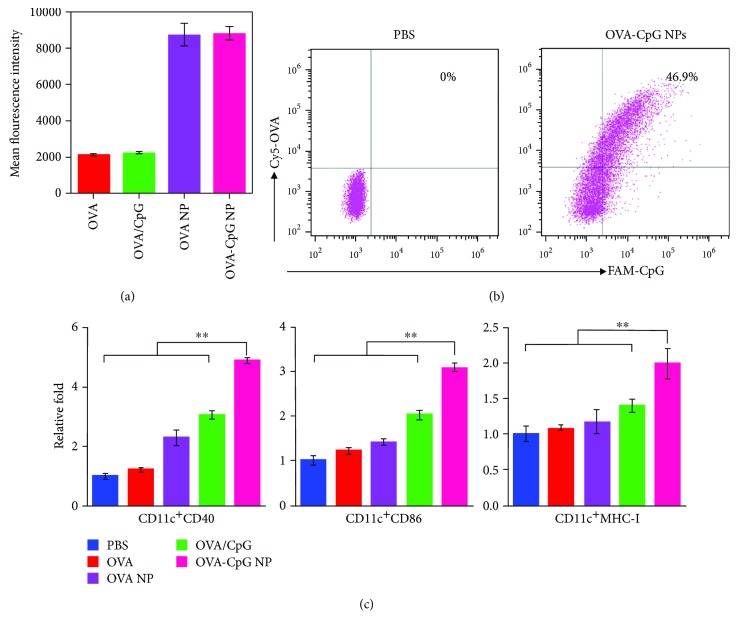
Comparison of DC uptake and maturation with different vaccine formulations in vitro. (a) Comparison of intracellular OVA amounts in DCs. (b) Representative cointernalization image of OVA and CpG in the PBS and OVA-CpG NP group. (c) Expression of recognition signals (SIINFEKL-MHC-I) and costimulatory markers (CD40 and CD86) in DCs after incubation with different vaccine formulations. The bars represent sd (*n* = 3). Statistical significance was defined as ^∗∗^ *P* < 0.01.

The prerequisite for potent T cell activation is the efficient utilization of antigen and adjuvant by APCs. In this respect, dendritic cells (DCs), the most professional APCs, were harvested to evaluate the uptake of OVA and CpG in vitro. As shown in Figure 3(a), the mixture with CpG (OVA/CpG) had a very small effect on the OVA internalization. Owing to particulate formulation and high loading efficiency, the uptake amount of OVA was significantly improved by 4-fold in the OVA NP group (self-assembled OVA and inactive oligodeoxynucleotide). Such superior uptake could be mostly maintained once DCs were treated with OVA-CpG NPs (Figures S8 and S9). Meanwhile, the assembled CpG was simultaneously internalized (verified by the scatter FCM data in Figure 3(b)).

Next, we evaluated the DC maturation after treatment with different formulations (Figure 3(c)). Although OVA NPs facilitated the DC uptake of OVA, a very small effect on SIINFEKL-MHC I (OVA-specific MHC-I) expression was observed. This result was acceptable, since exogenous antigen molecules alone are typically processed and presented with MHC-II for humoral immunity. Once CpG (a typical TLR-9 agonist) was mixed in (OVA/CpG), the SIINFEKL-MHC I expression was significantly enhanced due to the cross-presentation of antigen [24–27]. A further improvement was gained in the OVA-CpG NP group due to the increased uptake of both OVA and CpG. Similar results were obtained in costimulation. The expression of CD40 and CD86 gradually increased in the order of the PBS, OVA, OVA NP, OVA/CpG, and OVA-CpG NP groups, again demonstrating the superiority of OVA-CpG NPs in terms of DC maturation.

### 3.3. Lymph Node Targeting In Vivo

**Figure 4:**
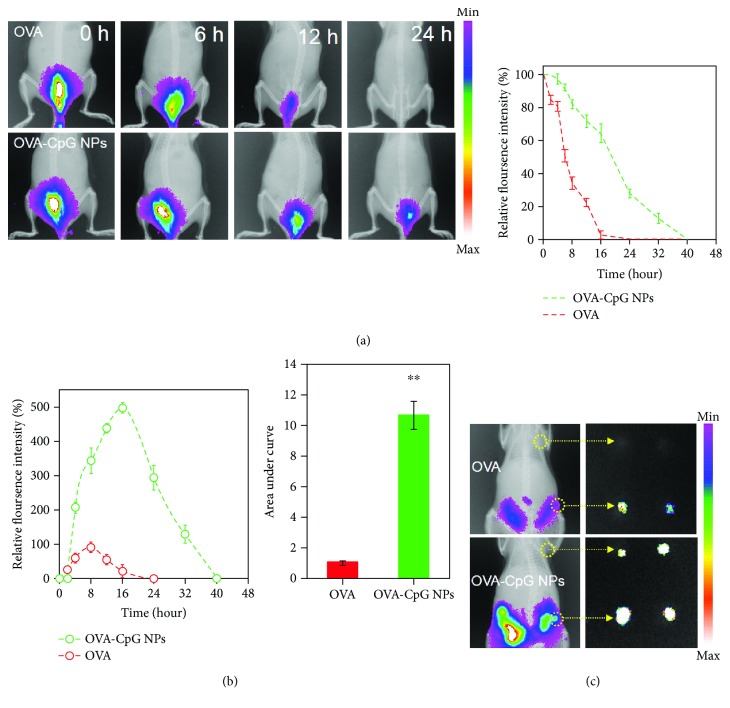
The clearance kinetics in situ and accumulation kinetics of different vaccine formulations in lymph nodes. (a) Mice were immunized with Cy5-OVA or Cy5-OVA-CpG NPs. Then, mice were subjected to fluorescence imaging of the injection site different time points, and the corresponding fluorescence intensity was quantified (right). (b) Quantitative sum fluorescence intensity of removed proximal lymph nodes at various time points. The relative fluorescence intensity was normalized to the peak fluorescence intensity in the Cy5-OVA group. The corresponding area under the curve represents the relative accumulated fluorescence intensity and was normalized to the accumulated fluorescence intensity in the Cy5-OVA group. (c) Representative fluorescence images of mice (lymph nodes are circled with a yellow dotted line) and harvested lymph nodes at 8 h (first line: distal LNs; second line: proximal LNs). The bars represent sd (*n* = 3).

Having demonstrated the enhanced APC uptake and maturation in vitro, we next assessed the ability of the vaccine to target lymph nodes in vivo. As the clearance behavior after vaccination highly correlated to subsequent transfer to lymph nodes, we monitored the clearance kinetics at the vaccination site. As shown in Figure 4(a), the near-infrared (NIR) fluorescence of free Cy5-OVA became very weak (less than 20%) at 12 h, which closed the time window for the transfer to lymph nodes. Once the OVA molecules were formulated into NPs, taking OVA-CpG NPs as an example, more than 30% of the signal remained at 24 h. Such an ameliorative kinetics behavior could supply more opportunities for NPs to target lymph nodes.

We also dissected the lymph nodes and determined their fluorescence signals to evaluate the performance of lymph node-targeting (Figure 4(b), Figure S10). In the first 2 h, no detectable signals were observed, suggesting neither free OVA nor OVA-CpG NPs arrived at the lymph node. Although the fluorescence intensity at the proximal lymph node area gradually increased over time in both groups, distinct kinetics and intensities were observed. In detail, the signals peaked at 8 h and 16 h for OVA and OVA-CpG NPs, respectively. Meanwhile, the peak intensity of OVA-CpG NPs was ~5 times higher than the value of free OVA. As a result, the area under the curve (AUC) of OVA-CpG NPs was significantly improved by 10-fold. More importantly, the distal lymph nodes were also bright in the OVA-CpG NP group (Figure 4(c)), suggesting a good capacity for long-distance delivery. In contrast, free OVA failed to transfer to distal lymph nodes due to the rapid clearance. This superior lymph node targeting of OVA-CpG NPs paved the way for subsequent immune responses.

### 3.4. Immune Response in Lymph Nodes

**Figure 5:**
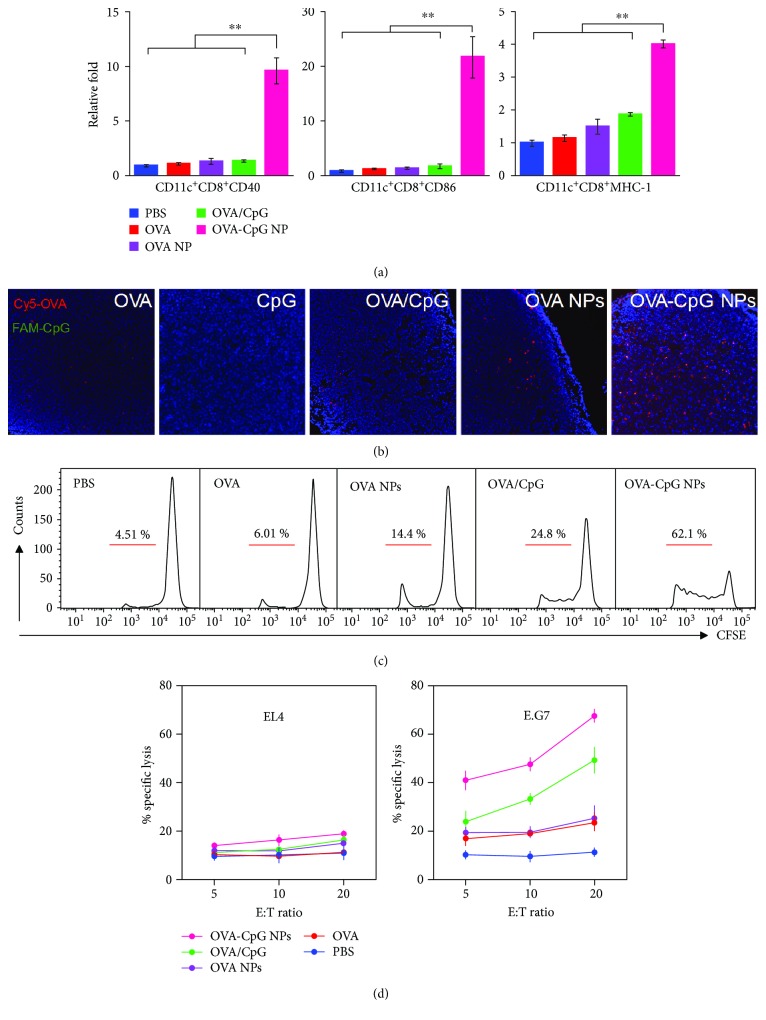
Immune response in lymph nodes with different vaccination formulations. (a) Expression of recognition signals (SIINFEKL-MHC-I) and costimulatory markers (CD40 and CD86) in CD8^+^ DCs at 24 h after immunization. (b) The distributions of OVA and CpG in lymph nodes at 8 h after immunization with different vaccine formulations (OVA was labeled with Cy5 and is represented in red; CpG was labeled with FITC and is represented in green; nuclei were labeled with DAPI and are represented in blue; and the colocalization of OVA and CpG is shown in orange). (c) In vivo proliferation of OVA-specific T cells at 24 h after immunization. (d) In vitro killing assay showing the percentage of specific lysis at different effector/target (E : T) cell ratios. The bars represent sd. Statistical significance was defined as ^∗∗^ *P* < 0.01.

The above results prompted us to evaluate the immune response in lymph nodes. Among DC populations, CD8^+^ DCs have been considered the main cells responsible for cross-presentation of exogenous antigens [28, 29]. Therefore, we investigated their maturation (Figure 5(a)). The expression of costimulators (CD40 and CD86) and SIINFEKL-MHC I at 24 h increased in the order of the PBS, OVA, OVA NP, OVA/CpG, and OVA-CpG NP groups. The CD8^+^ DC maturation was the best in the OVA-CpG NP group probably because of their superior codelivery of OVA and CpG to lymph nodes (Figure 5(b)). In contrast, the free or mixed components in other groups failed to efficiently drain to lymph nodes and be captured by DCs vastly (Figure S11), which led to varying degrees of compromise of the CD8^+^ DC maturation. In contrast with that an in vitro experiment, OVA-CpG NPs exhibited more superiority performance on DC activation in vivo experiment, which could be mainly attributed to two aspects. On the one hand, much more antigen and CpG drained to lymph nodes in the OVA-CpG NP group compared with those in other groups (Figure 5(b)). In this case, DCs in lymph nodes captured ~9-fold of antigen in the OVA-CpG NP group compared with that in the OVA/CpG group (Figure S9). However, such a significance was compromised to ~4.5 in vitro experiment (Figure 3(a)), since DCs were exposed to the same amount of antigen and CpG in the cell culture system. On the other hand, CD8^+^ DC in lymph nodes was a professional DC subset for cross-presentation. Once this type of DC was activated, the expression of CD40, CD86, and MHC-I was much higher than that in other subsets of DC.

The CD8^+^ DC maturation in lymph nodes facilitated their activation of adjacent CD8^+^ T cells [30]. As shown in Figure 5(c), OVA alone failed to induce the proliferation due to the rapid clearance. Although the activation was gradually ameliorated in the OVA NP and OVA/CpG groups, the percentage of proliferated CD8^+^ T cells in each of these groups was below 25%. As expected, 62.1% of CD8^+^ T cells had proliferated in the OVA-CpG NP group, indicating the most potent activation. Generally, during the first 24 hours of stimulation in most previous studies, CD8^+^ T cells prepared for clonal expansion and increase in size. Soon after, CD8^+^ T cell division commenced at a rapid rate (~6–8 hours per cell division). So the number of CD8^+^ T cells peaked at days 3 to 7 after immunization [31–35]. However, it is worth mentioning the proliferation of CD8^+^ T cells occurred at 24 h after the immunization with OVA-CpG NPs. A possible explanation to such a distinguishing kinetics could be attributed to the fate of vaccine formulations in vivo. As the OVA-CpG NPs quickly drained to lymph nodes at 2–4 h, CD8^+^ T could be activated by the resident DCs in lymph nodes rapidly, rather than the conventional homed APCs. In this case, the time of activation could be significantly shorten. Such an expeditious immune response owing to the efficient lymph node targeting will be favorable in fighting against acute infection in the clinic.

For further verification, we also carried out a specific lysis assay to evaluate the cytotoxicity of these proliferated CD8^+^ T cells (Figure 5(d)). Administration of OVA alone showed little effect on the cytotoxic lysis of E.G7 cells (an OVA-expressing derivative of EL4 cells). In line with the aforementioned data, the lysis effect on E.G7 cells was gradually enhanced in the OVA NP and the OVA/CpG groups. Notably, up to 70% of E.G7 cells were lysed in the OVA-CpG NP group, whereas no evident cytotoxicity to EL4 cells was observed. Such an effective and specific aggressivity against antigen-positive targets supports the potential application of OVA-CpG NPs as safe and high-performance nanovaccines for cytotoxic T cell activation.

## 4. Conclusion

We invented a novel and simple nanotechnology to prepare assembled antigen and adjuvant nanoparticles with high loading efficiency. The nanoparticles were assembled without carriers due to the electrostatic interaction between antigens and CpG. This particulate formulation enhanced APC uptake and maturation. After vaccination, antigen and adjuvant were quickly and efficiently transported to proximal and distal lymph nodes, which improved the antigen and adjuvants utilization and enhanced the cross-presentation of antigen. Then, an effective and quick immune response was aroused in lymph nodes in the form of activating CD8^+^ DCs and specific T cells at 24 h after immunization. So the nanoparticles we constructed exhibited great potential as therapeutic vaccines. Hence, the real performance of OVA-CpG NPs at the animal level, even in tumor models, will be evaluated in upcoming work. Meanwhile, based on the assembly technology we developed, other combinations, such as tumor antigens, poly-IC, and siRNA, all could be constructed to promote the development of other nanodelivery systems to cater to specific applications.

## Data Availability

All data arising from this study are contained within the manuscript and supplementary information files.

## Supplementary Materials

Supplementary 1 Figure S1: the photographs of water, nanoparticles before ultracentrifugation, and precipitation after ultracentrifugation.

Supplementary 2 Figure S2: circular dichroism of OVA in water and GSH.

Supplementary 3 Figure S3: size distribution of OVA.

Supplementary 4 Figure S4: zeta potential of OVA in different solutions.

Supplementary 5 Figure S5: zeta potential of CpG in different solutions.

Supplementary 6 Figure S6: influence of OVA and CpG concentrations on the diameter and yield of OVA-CpG NPs.

Supplementary 7 Figure S7: CLSM images of Cy5-OVA-FAM-CpG NPs.

Supplementary 8 Figure S8: the percentages of OVA+ cells in DCs.

Supplementary 9 Figure S9: time-dependent uptake of OVA/CpG and OVA-CpG NPs in DC.

Supplementary 10 Figure S10: the representative fluorescence images of proximal lymph nodes at different time points in OVA-CpG NP and OVA groups.

Supplementary 11 Figure S11: the internalization of different formulations in DC of lymph nodes.
