# Supplementary figures and images for: Lymph Node-Targeting Nanovaccine through Antigen-CpG Self-Assembly Potentiates Cytotoxic T Cell Activation
Source: J Immunol Res. 2018 Jun 19;2018:3714960. doi: 10.1155/2018/3714960 (PMC6029500; doi:10.1155/2018/3714960)

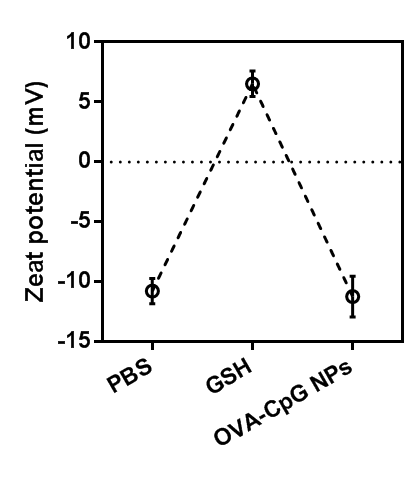

Supplement: Supplementary 4 — Figure S4: zeta potential of OVA in different solutions. [file 3714960.f4.docx]

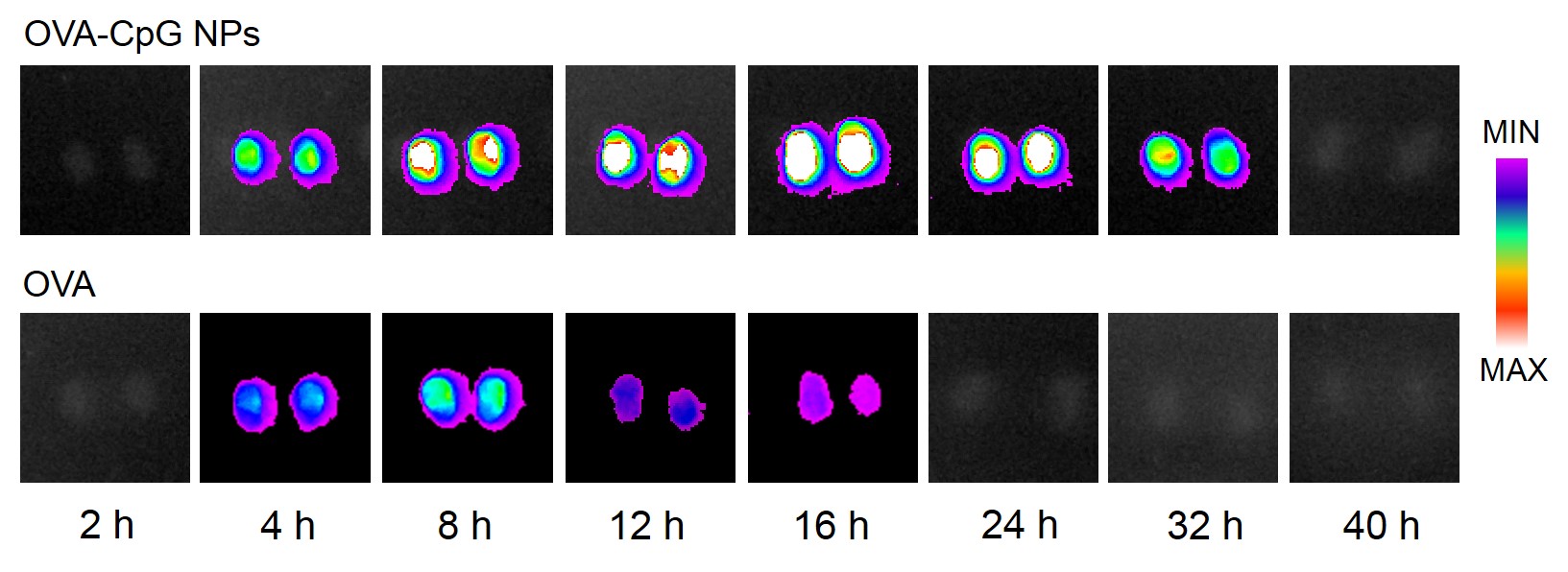

Supplement: Supplementary 10 — Figure S10: the representative fluorescence images of proximal lymph nodes at different time points in OVA-CpG NP and OVA groups. [file 3714960.f10.jpg]
