# Supplementary material for: Lymph Node-Targeting Nanovaccine through Antigen-CpG Self-Assembly Potentiates Cytotoxic T Cell Activation
Source: J Immunol Res. 2018 Jun 19;2018:3714960. doi: 10.1155/2018/3714960 (PMC6029500; doi:10.1155/2018/3714960)
Supplement: Supplementary file 3 [file 3714960.f11.docx]

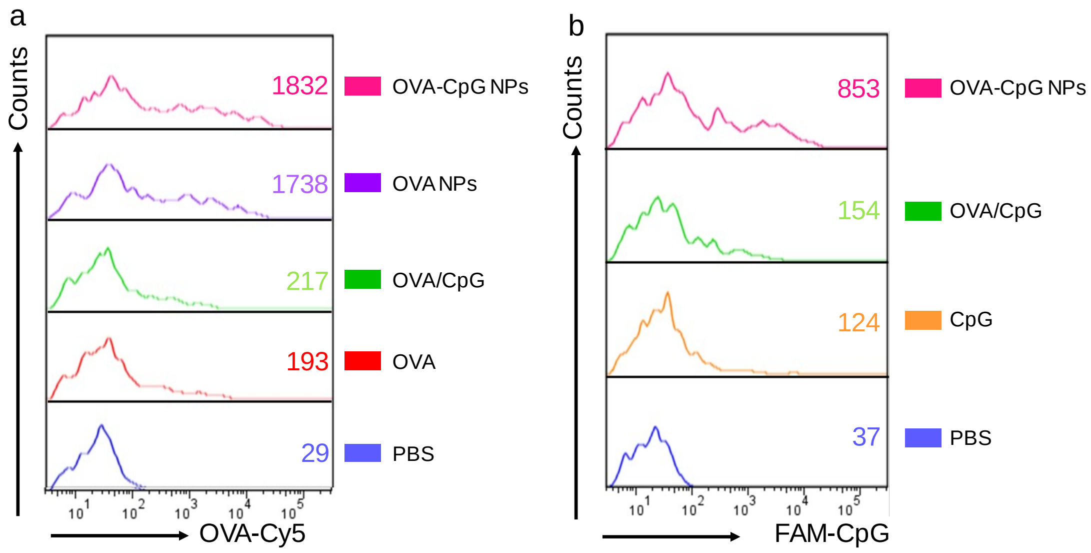
a
Counts
1832
OVA-CpG NPs
1738
OVA NPs
217
OVA/CpG
193
OVA
29
PBS
OVA-Cy5
b
Counts
853
OVA-CpG NPs
154
OVA/CpG
124
CpG
37
PBS
FAM-CpG
