# Supplementary material for: Lymph Node-Targeting Nanovaccine through Antigen-CpG Self-Assembly Potentiates Cytotoxic T Cell Activation
Source: J Immunol Res. 2018 Jun 19;2018:3714960. doi: 10.1155/2018/3714960 (PMC6029500; doi:10.1155/2018/3714960)
Supplement: Supplementary file 4 [file 3714960.f2.docx]

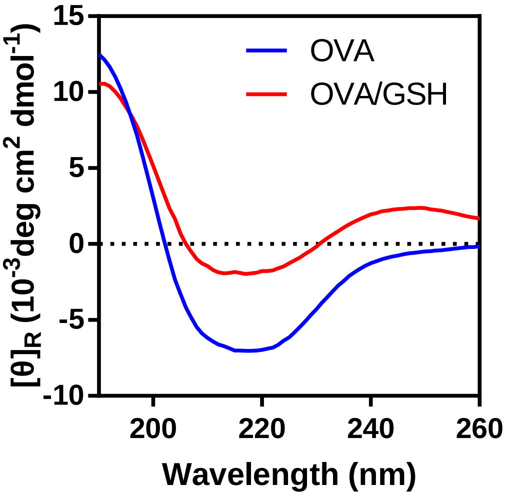

OVA
OVA/GSH
[θ]R (10-3deg cm2 dmol-1)
15
10
5
0
-5
-10
200
220
240
260
Wavelength (nm)
