# Supplementary material for: Lymph Node-Targeting Nanovaccine through Antigen-CpG Self-Assembly Potentiates Cytotoxic T Cell Activation
Source: J Immunol Res. 2018 Jun 19;2018:3714960. doi: 10.1155/2018/3714960 (PMC6029500; doi:10.1155/2018/3714960)
Supplement: Supplementary file 6 [file 3714960.f5.docx]

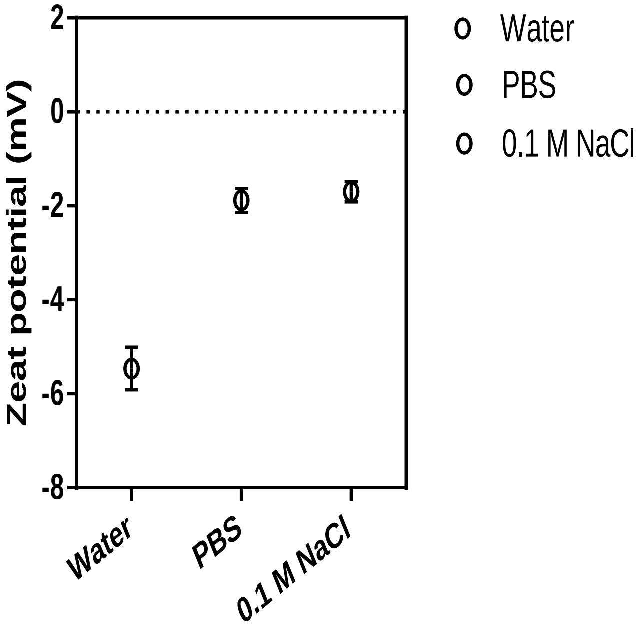
Zeat potential (mV)
2
0
-2
-4
-6
-8
Water
PBS
0.1 M NaCl
Water
PBS
0.1 M NaCl
