# Supplementary material for: Lymph Node-Targeting Nanovaccine through Antigen-CpG Self-Assembly Potentiates Cytotoxic T Cell Activation
Source: J Immunol Res. 2018 Jun 19;2018:3714960. doi: 10.1155/2018/3714960 (PMC6029500; doi:10.1155/2018/3714960)
Supplement: Supplementary file 7 [file 3714960.f6.docx]

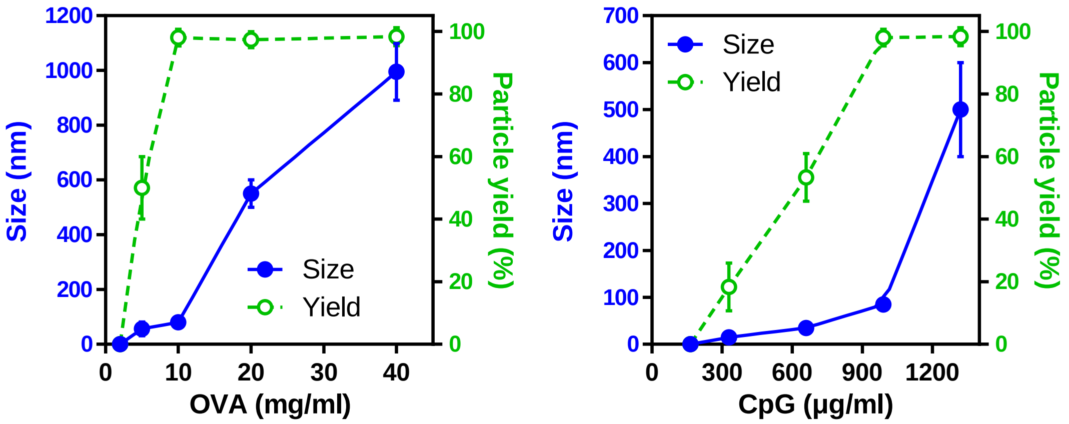

Size (nm)
Particle yield (%)
OVA (mg/ml)
Size
Yield
0
200
400
600
800
1000
1200
0
20
40
60
80
100
0
10
20
30
40
Size (nm)
Particle yield (%)
CpG (μg/ml)
Size
Yield
0
100
200
300
400
500
600
700
0
20
40
60
80
100
0
300
600
900
1200
