# Supplementary material for: Lymph Node-Targeting Nanovaccine through Antigen-CpG Self-Assembly Potentiates Cytotoxic T Cell Activation
Source: J Immunol Res. 2018 Jun 19;2018:3714960. doi: 10.1155/2018/3714960 (PMC6029500; doi:10.1155/2018/3714960)
Supplement: Supplementary file 9 [file 3714960.f8.docx]

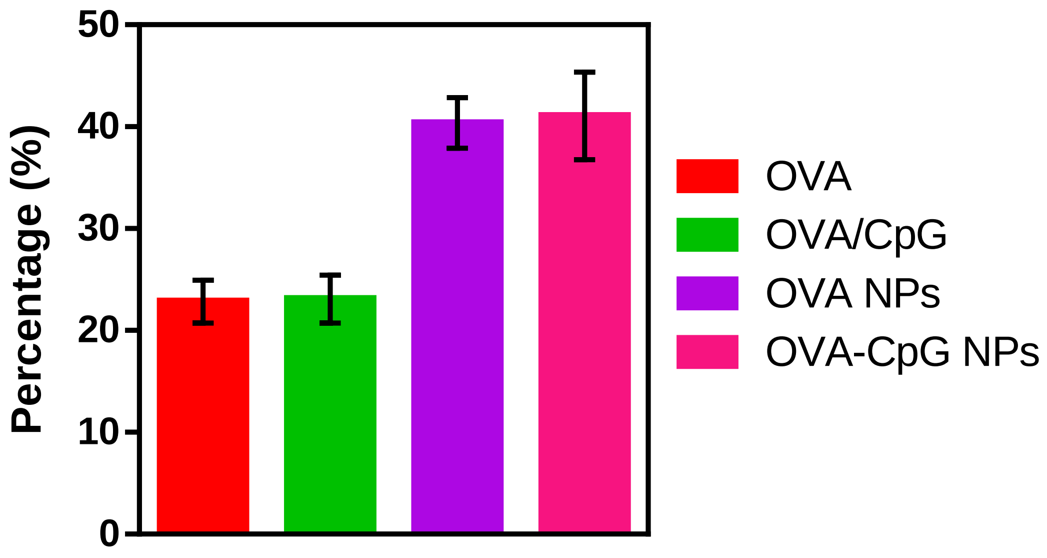
50
40
30
20
10
0
Percentage (%)
OVA
OVA/CpG
OVA NPs
OVA-CpG NPs
