# Supplementary material for: Lymph Node-Targeting Nanovaccine through Antigen-CpG Self-Assembly Potentiates Cytotoxic T Cell Activation
Source: J Immunol Res. 2018 Jun 19;2018:3714960. doi: 10.1155/2018/3714960 (PMC6029500; doi:10.1155/2018/3714960)
Supplement: Supplementary file 10 [file 3714960.f9.docx]

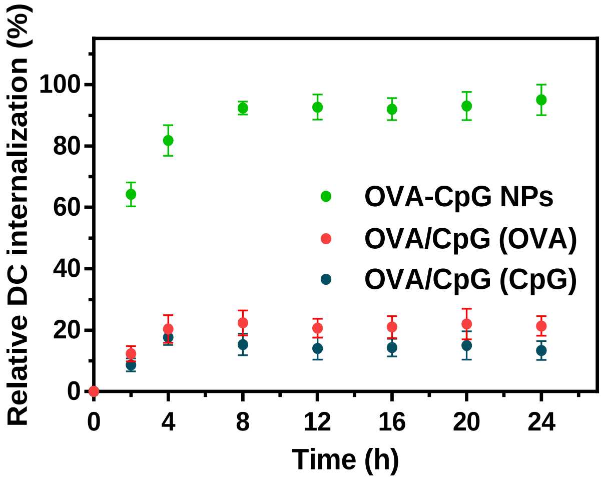

Relative DC internalization (%)
100
80
60
40
20
0
0
4
8
12
16
20
24
Time (h)
OVA-CpG NPs
OVA/CpG (OVA)
OVA/CpG (CpG)
